# Carob fruit extract as naturally products corrosion inhibitor for copper-nickel alloys in brine solutions

**DOI:** 10.1038/s41598-024-80589-7

**Published:** 2024-11-26

**Authors:** Abd El Aziz S. Fouda, Mona Nageeb, Ghalia A. Gaber, Amal S. Ahmed, Ahmed A. El-Hossiany, Mohamed F. Atia

**Affiliations:** 1 Chemistry Department, Faculty of Science, Mansoura University, Mansoura, 35516 Egypt; 2 Department of Chemistry, Faculty of Science (Girls), Al-Azhar University, Nasr City, Cairo, 11754 Egypt; 3 Institute of Aviation Engineering and Technology, Cairo, Egypt

**Keywords:** Corrosion, Biomaterials

## Abstract

Copper-nickel alloys are the preferred material for desalination facilities and condensers and heat exchangers that use saltwater as a coolant. The eco-friendly compounds especially Carob fruit extract (CFE) has emerged as excessive green corrosion inhibitor for alloys. Cu-Ni alloys are widely used in various industries due to their excellent corrosion resistance. However, their performance can be compromised in aggressive environments like seawater (which is approximately 3.5% NaCl). To evaluate the corrosion behavior of these alloys and the effectiveness of corrosion inhibitors, researchers often employ weight loss, potentiodynamic polarization, and impedance spectroscopy techniques. The results showed that CFE exhibited a good ability to decrease the CR of alloys in 3.5% NaCl solution. The inhibition efficacy (IE) was reached to 92.6% and ~ 83.2% at 300 ppm dose of CFE for Cu-10Ni alloy and Cu-30Ni alloy, respectively. The CR increases with temperature rising, but the addition of CFE reduces the CR, and the reduction depends on the dose of the extract. Adsorption of the extract gives a good fit to Langmuir, Temkin, and Freundlich isotherms model. The free adsorption energies of CFE on Cu-10Ni and Cu-30Ni alloys were 17.61 and 15.86 kJ mol^−1^, respectively, suggesting that CFE was weakly held to both alloys. The presence of a protective film on the alloys surface is confirmed by using scanning electron microscopy (SEM), energy dispersive X-ray (EDX) spectroscopy and atomic force microscope (AFM). The study suggests that utilizing affordable, natural substances as green corrosion inhibitors presents a new strategy for promoting both resource efficiency and environmental sustainability.

**Supplementary Information:**

The online version contains supplementary material available at 10.1038/s41598-024-80589-7.

Copper-nickel alloys, such as the engineering grades 90 − 10 and 70 − 30, are widely used in various industries due to their desirable properties. These alloys exhibit excellent mechanical workability, strong corrosion resistance and high thermal conductivity, making them ideal for applications. Copper-nickel alloys are widely used in marine industries due to their exceptional corrosion resistance in seawater environments. These alloys are particularly valuable in applications like thermal desalination, offshore oil and gas, power generation, and commercial and naval shipping. Their durability and resistance to corrosion make them essential for ensuring the long-term reliability and safety of marine infrastructure. Additionally, these alloys find applications in hydraulic tubing, including brake tubing^1–3^. It is liable to several types of corrosion, including pitting corrosion brought on by various corrosive species, such as ions of nitrate, sulfate, and chloride. There have been reports of several Cu-Ni alloys corroding in chloride solutions and in seawater under various conditions^4^. Depending on the alloy composition, there have been contradictory findings about the predominance of selective electro-dissolution of nickel and copper. Copper-nickel can be strengthened by adding aluminum through standard precipitation hardening processes. To obtain desirable qualities, copper-nickel is frequently mixed with small amounts of other elements. Weldability and hot workability, however, may be hampered by certain impurity elements, such as lead, sulfur, carbon, bismuth, antimony, and phosphorus. Chemical doses must therefore be strictly regulated^5,6^. The use of inhibitors is one of the most crucial strategies for protecting copper alloys against corrosion. It involves the use of chemical substances that, when added in small amounts to a harsh environment, can lessen the exposed metal’s tendency to corrode^7^. “Plant extracts are a popular choice for corrosion inhibitors due to their affordability and environmental friendliness. These natural substances, derived from renewable resources, are readily available, non-toxic, and considered eco-friendly options for corrosion prevention^8–12^. Plant extracts can inhibit corrosion due to the presence of heterocyclic compounds like alkaloids, flavonoids, carbohydrates, and proteins. These organic molecules often have polar groups with nitrogen, sulfur, or oxygen atoms, as well as triple or conjugated double bonds with aromatic rings. This structure allows them to adhere to metal surfaces, a crucial mechanism for corrosion prevention^13–15^. Fouda et al.^16^ used various electrochemical techniques to investigate how enamino nitrile derivatives affect the adsorption and inhibition of corrosion on a Cu10Ni alloy in 0.5 M HCl. The inhibition efficiency increased as we added more of these derivatives but decreased when the temperature rose. Chieb et al.^17^ We studied the effects of 3-methyl-4-amino-1,2,4-triazole on copper-nickel 70 − 30 (Monel) corrosion in 3% NaCl solution at room temperature. Using various methods, we found that increasing the dosage to 100 parts per million (ppm) significantly improved corrosion inhibition from 20–60–95%. “Further increasing the dosage to 100 ppm resulted in a slight decrease in inhibition to 85%.” Hmamou et al.^18^ studied carob seed oil (CO) as a corrosion inhibitor for C38 steel in 1 M hydrochloric acid (HCl). We found that CO’s effectiveness in preventing corrosion depends on the amount used, reaching 86.7% at a concentration of 0.5 grams per liter. We also examined the electrochemical and theoretical behavior of carbon steel in 1 M HCl solution using carob (Ceratonia Siliqua L.) seed extract (CFE)^19^. According to the results, CFE extract has a respectable 95% inhibitory efficacy against corrosion at 100 mg/l at 298 °K. Ghazi et al.^20^ investigated the Corrosion Inhibition of Carob Pod Pulp (Ceratonia Siliqua L.) on Carbon Steel Surface C38 in Hydrochloric Acid. The obtained inhibition data showed that the Gallic acid aqueous extract exhibited good anticorrosion action, with an inhibition rate of 91.32% at 3 g/l at 323 K. Fouda et al.^21^ investigated the effect of Ceratonia silica extract on the corrosion of copper and brass in aqueous 1 M nitric acid. When the inhibitor dose is increased, the value of inhibition efficiency rises to 82.8% and 97% for Cu and α-brass, respectively, and falls when the temperature does. Gaber et al.^22^ describe the use of green tea aqueous extract as a sustainable corrosion inhibitor for two Cu-Ni alloys in 3.5% NaCl. Some plant extracts were used as corrosion inhibitors for metals in NaCl medium were shown in Table 1 below.

**Table 1:** Lists of plant extracts used as corrosion inhibitors for metals and alloys in NaCl solutions.

Source	Metal/medium	IE (%)	Ref.
*Ocimum basilicium seed extract*	2024 Al alloy 3.5% NaCl	95.5% at 1 g/L.	^23^
*pumpkin seeds extract*	Al alloy, 3.5% NaCl	95% at 1 g/L	^24^
*Linum usitatissimum seeds extract*	AA2024 and NaCl (3.5%)	65 to 82% 80 at 1200 ppm	^25^
*Mansoa alliacea extract*	Zn in NaCl 3%	92% at 300 ppm	^26^
*Hibiscus sabdariffa (Roselle) extract*	mild steel in 3.5% NaCl	90.26%, 8.561 × 10^− 4^ M	^27^
*Jatropha curcas extract*	mild steel in 3.5% NaCl	81.32% at 1.205 × 10^− 4^ M	^27^
*olive (Olea europaea L) leaf* extract	steel surface, brine solution	83%, at 50 ppm	^28^
*Catharanthus roseus*,* Laurus nobilis*,* Areca catechu extracts*	mild steel in 0.5 M NaCl	82%, 65%, 50% at 2000 ppm, respectively	^29^
*Persian Liquorice extract*	mild steel in NaCl	98.8% at 600 ppm	^30^
*Ocimum basilicium seeds extract*	Al in 3 wt% NaCl	95.3%, at 1 g / L	^31^
*Carob fruit extract*	Cu-10Ni, Cu-30Ni alloys in3.5% NaCl	92.6% and ~ 83.2% at 300ppm, respectively	Our results

The findings demonstrate that the tested extract had a good ability to reduce the rate at which alloys corroded in 3.5% NaCl. Prior research on Ceratonia Siliqua L., or carob, has identified a number of components, including minerals, proteins, carbohydrates, polyphenols, and flavonoids”. This study is innovative in that it uses an extract from the fruit of Carob as a corrosion inhibitor to increase the resistance of Cu-Ni alloys to 3.5% M NaCl solution. The WL technique and electrochemical experiments have been used to determine the extract’s protective effect. Moreover, atomic force microscopy (AFM), scanning electron microscopy (SEM), and energy-dispersive X-ray analysis (EDX) were used to investigate the surface’s shape.

## Experimental

### Materials and solutions

We used two types of “Cu-Ni alloys in our experiments: 90/10 Cu-Ni and 70/30 Cu-Ni. The 90/10 alloy contained 1.67% iron, 0.768% manganese, 9.27% nickel, and the rest copper. The 70/30 alloy had 0.02% iron, 0.44% silicon, 0.51% calcium, 29.16% nickel, and the rest copper. The coupon samples were cleaned with acetone, twice-distilled water before each experiment. They were then dried. In an effort to reduce experimental mistakes, this was also completed prior to measurements. The methanol (BDF) extract from the fruit Ceratonia Siliqua L is the inhibitor. NaCl (3.5%) solution (Sigma Aldrich)” combined with bidistilled water makes up the caustic solution.

Solution is the Carob fruits which was selected and purchased from El-Niqaty store at Mansoura City, Egypt. “The Carob fruits were identified at the Botanical herbarium at Sadat City University. We air-dried and ground the plant sample into a fine powder. Methanol is a good solvent for extracting plant compounds, so we soaked 100 grams of the powder in 300 milliliters of 70% methanol for 48 hours at room temperature^32^. We then removed and dried the extract using a rotary evaporator. The concentrated extract was the crude extract we studied. We dissolved the extract in ethanol (1 gram per liter) and stored it in the refrigerator at 4 degrees Celsius^33^. We used Fourier Transform Infrared (FT-IR) spectroscopy to identify the main functional groups in the extract before and after studying its corrosion inhibition properties. To study corrosion inhibition, we prepared solutions with different concentrations of carob fruit extract (50, 100, 200, and 300 parts per million) by diluting a stock solution (1000 parts per million) with distilled water”. We compared these solutions to a control solution without any inhibitor. All solutions were made fresh using high-quality reagents and distilled water.

### Corrosion studies

We used chemical and electrochemical methods to evaluate how carob, a natural product, affects the corrosion of Cu-Ni alloys in 3.5% sodium chloride (NaCl) solution. We tested the alloys with and without different amounts of carob.

### WL measurements

Measured corrosion loss using a simple method called gravimetric. “Cleaned and polished the samples, then soaked them in 50 milliliters of 3.5% sodium chloride (NaCl) solution with and without different amounts of carob (50, 100, 200, and 300 parts per million). We kept the samples at 25 degrees Celsius for 15 days and weighed them regularly. After the test, we cleaned the samples, dried them, and weighed them again”^34^. We conducted experiments in triplicate to ensure the reliability of our results. We conducted experiments in triplicate to ensure the reliability of our results. Equation (1)^35^ can be used to get the average weight reduction in grams:

1 $$\:\varDelta\:\:\text{W}={W}_{1}-{W}_{2}\:$$

The corrosion rate (CR) is evaluated as following (Eq. (2))^36^:

2 $$\:\text{C}\text{R}=\frac{\varDelta\:\text{W}\times\:\text{K}}{\text{A}\times\:\text{T}\times\:\text{D}}\:\:$$

The inhibition efficiency (IE) and surface coverage (θ) are determined by Eqs. (3) and (4):

3 $$\:\text{\%}\:\text{I}\text{E}\:=\frac{\text{C}{\text{R}}_{\text{o}}-\text{C}{\text{R}}_{\text{i}\text{n}\text{h}}}{\text{C}{\text{R}}_{\text{o}}}\:\:\times\:100\:\:\:\:\:\:\:\:\:\:\:\:\:\:\:\:\:\:\:\:\:\:\:\:\:\:\:\:\:\:\:\:\:$$

where $\:\text{C}{\text{R}}_{\text{o}}$ and $\:\text{C}{\text{R}}_{\text{i}\text{n}\text{h}}$ are the CR of alloys in the existence and lack of CFE natural products (as inhibitor), respectively. $\:{\uptheta\:}$ can be computed from Eq. (4)^37^

4 $$\:\text{T}\text{h}\text{e}\:\text{e}\text{x}\text{t}\text{e}\text{n}\text{t}\:\text{o}\text{f}\:\text{s}\text{u}\text{r}\text{f}\text{a}\text{c}\text{e}\:\text{c}\text{o}\text{v}\text{e}\text{r}\text{a}\text{g}\text{e}\:\left({\uptheta\:}\right)=\frac{\text{I}\text{E}}{100}\:\:\:\:\:\:\:\:\:\:\:$$

### PDP analysis

Electrodes composed of copper-nickel alloys were fabricated using epoxy cold resin mounting, Prepared samples of Cu-Ni alloys with exposed surfaces of one square centimeter. “Polished these surfaces with silicon carbide paper up to 2000 grit, cleaned them with acetone, and rinsed them with doubly distilled water. We conducted electrochemical experiments using a three-electrode cell setup, with a saturated calomel electrode as the reference, a platinum plate as the auxiliary electrode, and the Cu-Ni alloy as the working electrode. We observed how the open circuit potential (E_ocp_) of the alloy changed over time when immersed in a naturally aerated 3.5% sodium chloride solution at room temperature. We then performed potentiodynamic polarization experiments at a scan rate of 0.2 millivolts per second, scanning the potential within a range of E_ocp_ plus or minus 250 millivolts. We analyzed the resulting Tafel plots to determine the corrosion rate without extract (CR), with inhibitors (CR_inh_), and the corrosion potential (E_corr_). The inhibitive efficiency (% IE) and surface coverage (θ) were determined according to Eqs. 3 and 4. We performed electrochemical impedance spectroscopy (EIS) measurements at the open circuit potential in potentiostatic mode, applying a voltage perturbation of 10 millivolts over a frequency range of 100 kilohertz to 10 millihertz. We used fresh solutions and newly polished electrodes for all electrochemical experiments, preparing the electrodes with silicon carbide paper and using an electrochemical instrument (Voltalab40 Potentiostat PGZ301 Dynamic EIS Voltammetry)”. % IE and θ derived from the impedance tests are distinct by Eq. (5)^38^.

5 $$\:\:\:\:\:\:\:\:\text{\%}\:\text{I}\text{E}=\:\:{\uptheta\:}\:\text{x}\:100\:=\:\:\frac{\text{R}\text{c}\text{t}\left(\text{i}\text{n}\text{h}\right)-\text{R}\text{c}\text{t}}{\text{R}\text{c}\text{t}\left(\text{i}\text{n}\text{h}\right)}\:\:\times\:100\:\:\:\:\:\:\:\:\:\:\:\:\:\:\:\:\:\:\:\:\:\:\:\:\:\:\:\:\:\:\:\:\:\:$$

“R_ct_ and R_ct(inh)_ represent the charge transfer resistance lack and with the inhibitor, individually. The main parameters obtained from the Nyquist diagram are the charge transfer resistance R_ct_ (diameter of the high-frequency loop) and the double-layer capacitance C_dl_. According to electrochemical theory, the inverse of R_ct_ (1/R_ct_) is directly proportional to the double-layer capacitance”.

### Adsorption Isotherm

To get the adsorption isotherm, the extent θ of the two Cu-Ni alloys by the inhibitors (Carob fruit natural product) must be evaluated by diverse adsorption isotherms. In this study, we calculated θ for different doses of inhibitors in a marine solution using WL, PDP, and EIS measurements.

### Spectroscopic examination

We examined the surface morphology of the two Cu-Ni alloys with and without inhibitors. We prepared the alloy surfaces by grinding and polishing them before immersing them in the electrolyte solutions. After 15 days of immersion in NaCl solutions, we obtained scanning electron microscope (SEM) micrographs of the alloy surfaces. We used a JEOL scanning electron microscope equipped with energy dispersive spectroscopy (EDS).

## Results and discussion

### Impact of Carob natural products

**Fig. 1:**
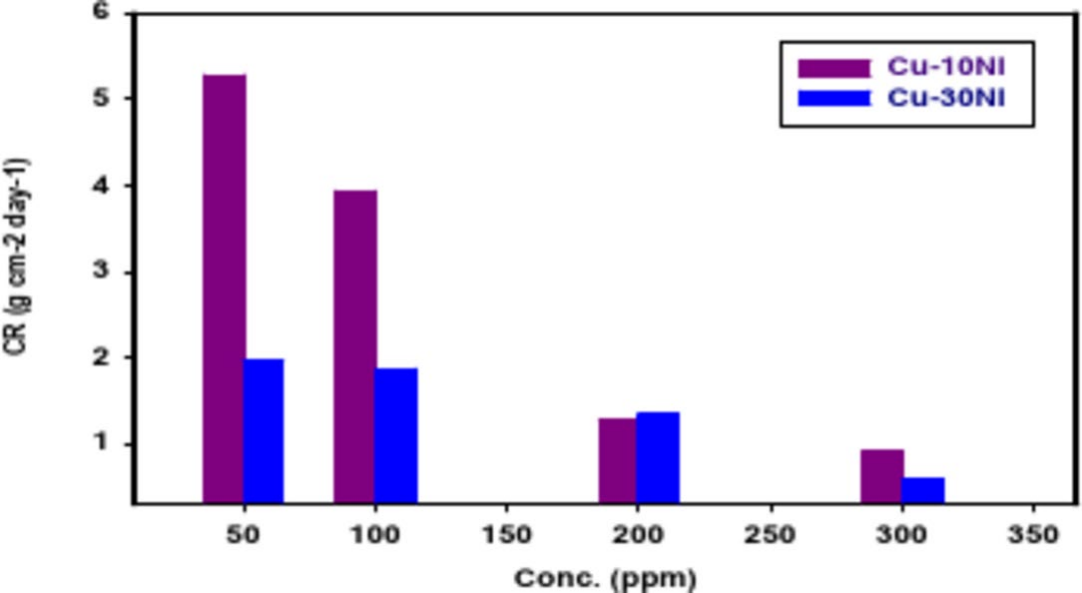
The variation of CR on two Cu-Ni alloys liquefaction in NaCl (3.5%) with altered doses of CFE at 298 K after 15 days engagement.

Table 2 and Fig. 1 summarize “CR and % IE values for two Cu-Ni alloys obtained using the weight loss technique with different concentrations of carob fruit extract (CFE). We observed that the weight loss of both alloys decreased in the presence of carob. Additionally, the corrosion rate decreased as the concentration of CFE increased. This effect might be due to a competition between carob’s ability to repair the passive film and the damage caused by aggressive ions. Our results demonstrate that carob effectively inhibits the corrosion of Cu-Ni alloys. The inhibition efficiency increased significantly with increasing carob concentrations, reaching a maximum of 83.2% for Cu-10Ni alloy and 92.6% for Cu-30Ni alloy at a concentration of 300 ppm. This behavior can be explained by the adsorption of carob extract components onto the surface of the Cu-Ni alloy, hindering the dissolution process and protecting the surface from the corrosive environment^39^. Increasing the concentration of carob in the corrosive solution further improves inhibition effectiveness, leading to the formation of a more durable and protective film on the alloy surface^40^. Thusly, we can infer that the Carob is a good inhibitor for Cu-Ni alloys in 3.5% NaCl solution. Table 2 demonstrates that alloy Cu-30Ni is superior to alloy Cu-10Ni in terms of corrosion inhibition. This is because, when exposed to seawater, corrosion product film is thick layer of cuprous oxide containing Ni^2+^/Ni^3+^ and Fe^2+^ forms on the surface beneath a thick layer of porous Cu (II) hydroxide/oxide”. This corrosion product film is what gives this alloy its exceptional resistance to corrosion in saltwater.

**Table 2:** % IE, CR, and θ for Cu-Ni alloys liquefaction in NaCl (3.5%) solution attendance and lack of altered doses of CFE following 15 days of immersion.

Conc., ppm	Cu-10Ni alloy			Cu-30Ni alloy		
	CR, (g cm^− 2^ day^− 1^)	(θ)	% IE	CR, (g cm^− 2^ day^− 1^)	(θ)	% IE
3.5% NaCl	3.5762	–	–	12.4017	–	–
50	1.9685	0.449	44.9	5.2822	0.574	57.4
100	1.8701	0.477	47.7	3.9371	0.683	68.3
200	1.3451	0.624	62.4	1.2795	0.897	89.7
300	0.6004	0.832	83.2	1.0186	0.926	92.6

### Effect of immersion time

**Fig. 2:**
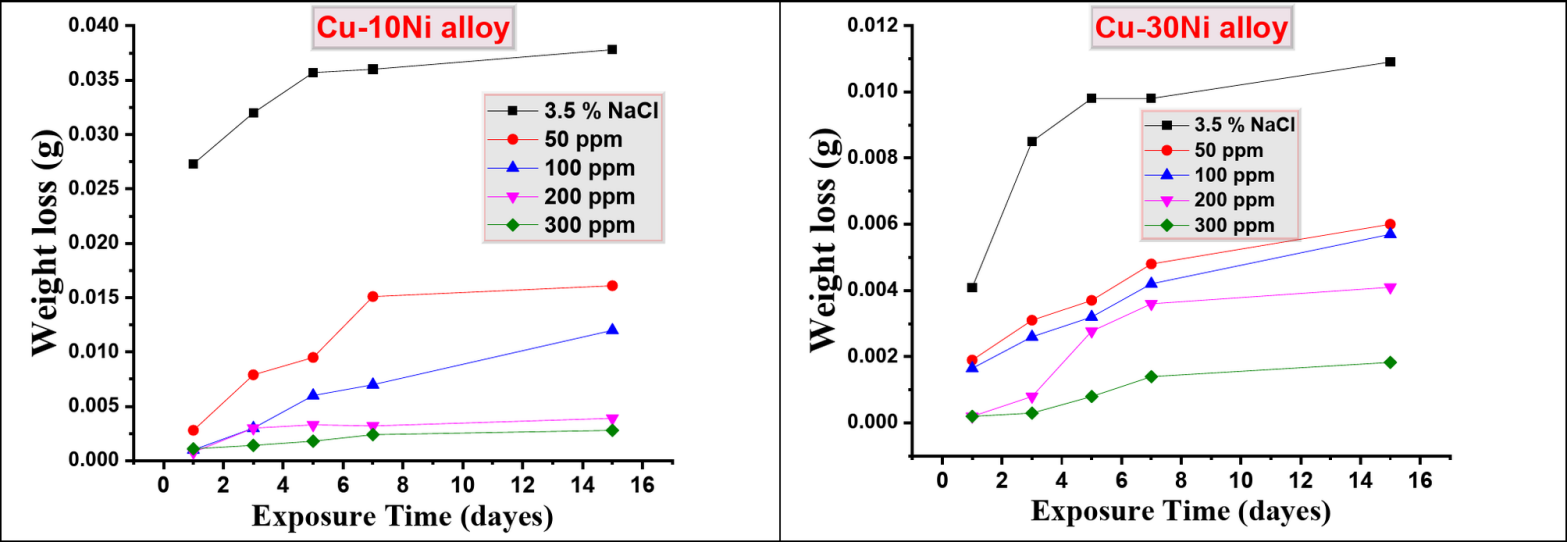
WL-time curves for Cu-Ni alloys dissolution in NaCl (3.5%) with and without different doses of Carob.

Figure 2 illustrates the change in weight loss of Cu-Ni alloys in 3.5% NaCl over time with and without different dose of CFE. “The graph shows that the weight loss (WL) increased with immersion time, but it was lower in the presence of the tested extract compared to the blank solution. The linear relationship between WL and time indicates that no insoluble surface oxide film formed during corrosion”. Furthermore, the carob fruit extract (CFE) was adsorbed onto the surface of the Cu-Ni alloys, hindering corrosion^40^.

### PDP measurements

**Fig. 3:**
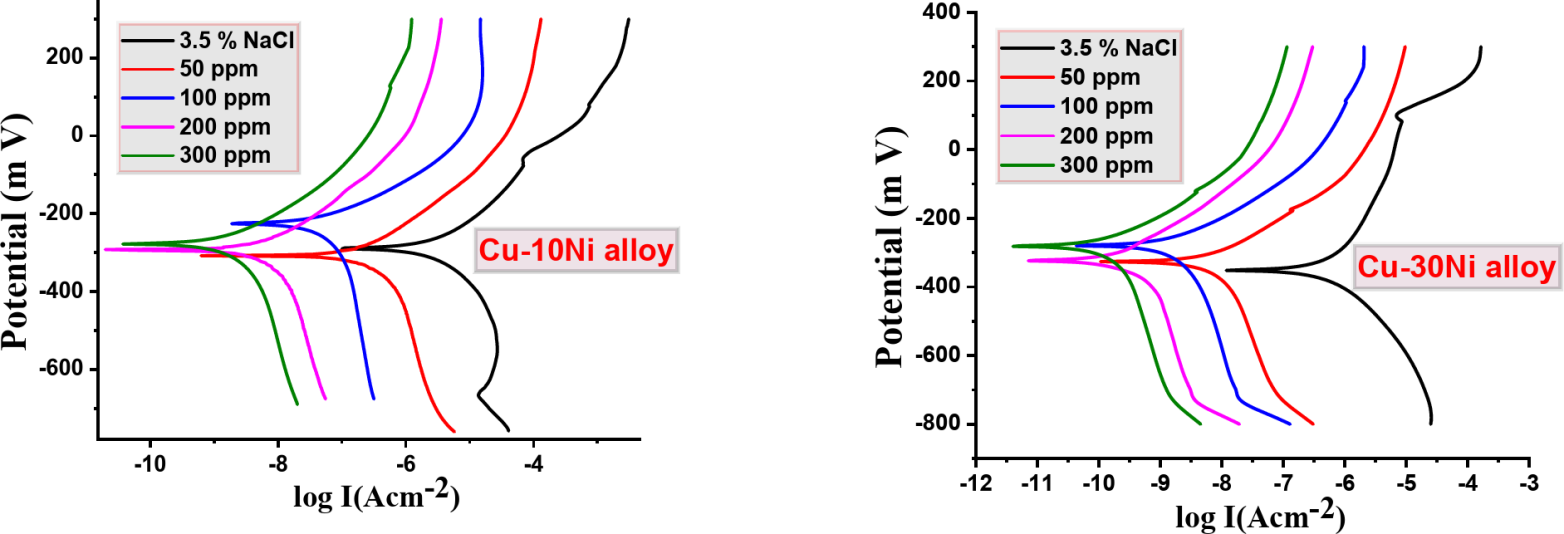
PDP bends for Cu-Ni alloys liquefaction in NaCl (3.5%) with and without different doses of CFE.

Figure 3 shows the PDP polarization bends for the two Cu-Ni alloys liquefaction in NaCl (3.5%) solution both with and without different doses of CFE. “In the uninhibited solution, the anodic curve initially shows an increase in corrosion current density (I_corr_), likely due to the dissolution of Cu-Ni alloys. However, this is followed by a plateau, indicating the formation of a passive layer of CuCl^41,42^. On the cathodic side, the primary reaction is the reduction of dissolved oxygen^43,44^. Adding CFE to the solution results in a clear shift of the corrosion potential (E_corr_) to more positive values. Additionally, as the CFE dose increases, I_corr_ decreases significantly, indicating a reduction in the corrosion rate. In this case, since the corrosion potential shift is less than 85 mV, CFE can be classified as a mixed-type inhibitor. This suggests that CFE adsorbs onto the Cu-Ni alloy surface, forming a protective barrier that effectively hinders the electrochemical corrosion process. Table 3 summarizes the relevant electrochemical parameters, including corrosion potential (E_corr_), corrosion current density (I_corr_), anodic (β_a_) and cathodic (β_c_) Tafel slopes, and inhibition efficiency (% IE), obtained from the polarization curves”. Additionally, the CR increases in the following order: Cu-30Ni < Cu-10Ni alloy.

**Table 3:** PDP parameters of CFE influence on the corrosion behavior of Cu-10Ni alloy in NaCl.

Alloy	Conc., ppm	-E_corr,_ mV vs. SCE	I_corr_ µA/cm^2^	β_a_ mV/dec	-β_c_ mV/dec	CR µm/y	% IE
Cu-10Ni alloy	3.5% NaCl	292.6	35.83	97.6	209.0	419.0	----
	50	313.3	18.39	110.7	138.8	215.0	48.68
	100	227.0	15.66	99.3	188.6	183.2	56.27
	200	299.2	13.35	39.6	62.8	156.1	62.74
	300	281.6	12.56	92.1	155.9	146.9	64.94
Cu-30Ni alloy	3.5% NaCl	358.3	25.378	122.3	157.1	296.8	----
	50	332.9	2.574	30.5	53.6	29.43	90.08
	100	285.8	2.323	79.5	157.4	27.17	90.84
	200	329.2	2.164	32.6	44.6	25.31	91.47
	300	287.6	1.065	31.5	51.1	11.87	96.00

### EIS analysis

**Fig. 4:**
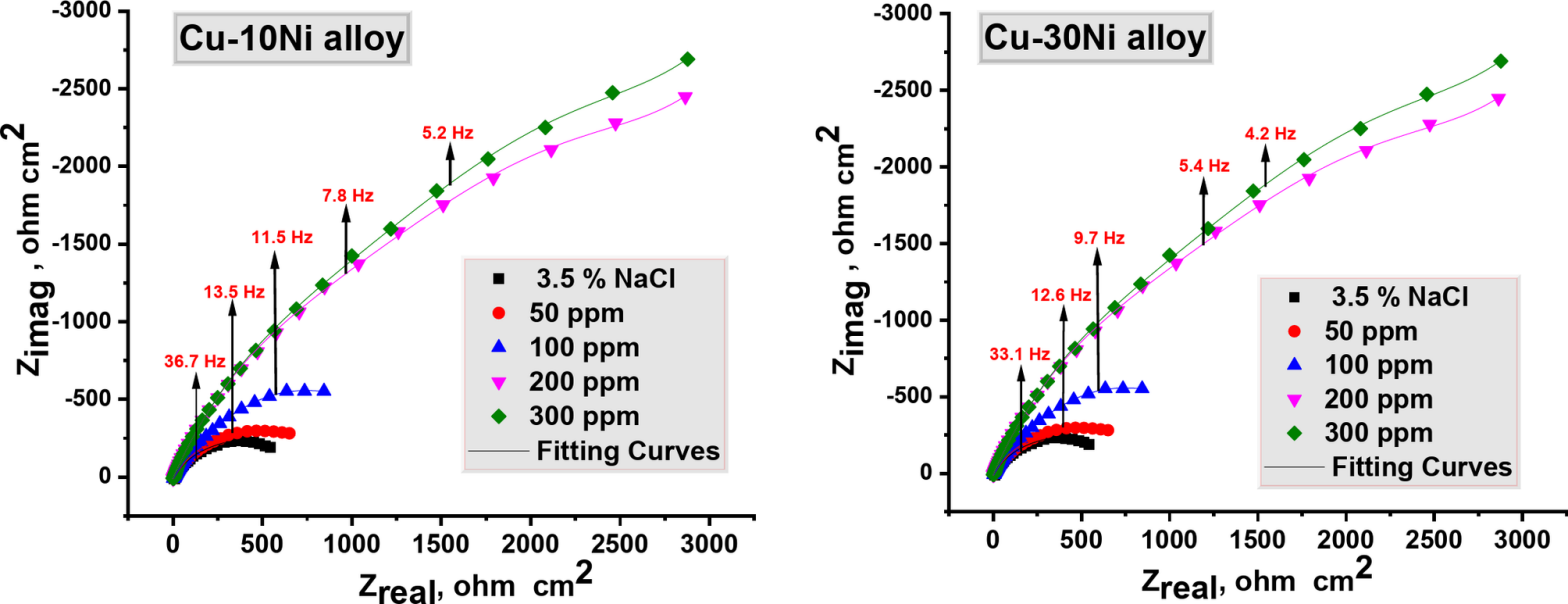
Nyquist bends for Cu-Ni Alloy liquefaction in NaCl 3.5% attendance and without of altered doses of Carob fruit extract, at 298 K.

To further investigate the inhibition process and confirm the results from the weight loss and potentiodynamic polarization experiments, “electrochemical impedance spectroscopy (EIS) studies on Cu-Ni alloys with and without different doses of carob fruit extract (CFE). Figure 4 shows the Nyquist plots for these experiments. Table 3 lists the impedance parameters, including polarization resistance (R_p_), double-layer constant phase element (CPE), surface coverage (θ), and inhibition efficiency (IE). The impedance spectra for Cu-Ni alloys in 3.5% NaCl solution exhibit a depressed semicircle with the center below the real axis. This behavior is typical for solid electrodes and is often attributed to surface roughness and other inhomogeneities. The Nyquist plots show a single semicircle that shifts along the real impedance (Z_r_) axis, indicating a single capacitive loop associated with the charge transfer process. As the CFE dose increases, the diameter of the loops also increases. This suggests that the protective layer formed on the electrode surface in the presence of the inhibitors is thicker and more effective at preventing ionic conduction^45^. The adsorption of extract molecules onto the metal surface displaces water molecules and other ions, reducing the electrical capacity of the surface. This modification increases the polarization resistance (R_p_), suggesting the formation of a protective layer on the Cu-Ni alloys. This layer acts as a barrier for mass and charge transfer. As shown in Table 4, Rp increases with increasing extract dose in 3.5% NaCl solution, indicating a significant anticorrosion effect of the CFE on Cu-Ni dissolution in 3.5% NaCl solution. The reciprocal of resistance is directly proportional to the corrosion current density (I_corr_) and corrosion rate (CR)^46^. As the extract dose increases, the corrosion rate (CR) decreases. Table 4 shows that the CPE values decreased with increasing extract doses in 3.5% NaCl solution. A decrease in CPE, which can be attributed to a decrease in the local dielectric constant or an increase in the thickness of the electrical double layer, suggests that the extract acts by adsorbing onto the alloy/solution interface”. The inhibition efficiency (IE) values calculated from EIS data are similar to those obtained from weight loss and potentiodynamic polarization measurements, indicating good agreement between the techniques.

**Table 4:** EIS data for Cu-Ni alloys in NaCl (3.5%) attendance and without of altered doses of CFE, at 298 K.

Conc. , ppm	Cu-10Ni Alloy			Cu-30Ni Alloy		
	*R*_*p*_ Ohm cm^2^	CPE mF/cm^2^	% IE	*R*_*p*_ Ohm cm^2^	CPE mF/cm^2^	% IE
3.5% NaCl	540.28	0.7652	----	1033.1	0.76977	----
50	873.37	0.8250	38.1	1955.0	0.7588	47.2
100	1733.5	0.7208	68.8	2576.2	0.7372	59.9
200	4577.3	0.7142	88.2	8873.9	0.72252	88.4
300	5202.1	0.7139	89.6	10286.0	0.71489	89.9

Comparison of electrochemical data with the conventional chemical data.

The WL method is often favored as it directly measures the extent of corrosion, eliminating the need for assumptions about the underlying chemical reactions. While electrochemical techniques offer higher % IE, this is attributed to their application on freshly prepared solutions.

### Adsorption isotherm

We calculated the surface coverage (θ) and inhibition efficiency (IE) from the weight loss (WL), potentiodynamic polarization (PDP), and electrochemical impedance spectroscopy (EIS) measurements using Eqs. 3 and 4. We analyzed the data by fitting it to various adsorption isotherms, including Langmuir, Temkin, and Freundlich. To understand the adsorption process of CFE on the Cu-Ni alloy surface, we graphically determined the surface coverage (θ) values from the different techniques using appropriate adsorption isotherm fitting. The best fit was obtained using the Langmuir adsorption isotherm, which is described by the following equation ^47^:

6 $$\:\frac{\text{C}}{{\uptheta\:}}=\frac{1}{{\text{K}}_{\text{a}\text{d}\text{s}}}+\text{C}$$

**Fig. 5:**
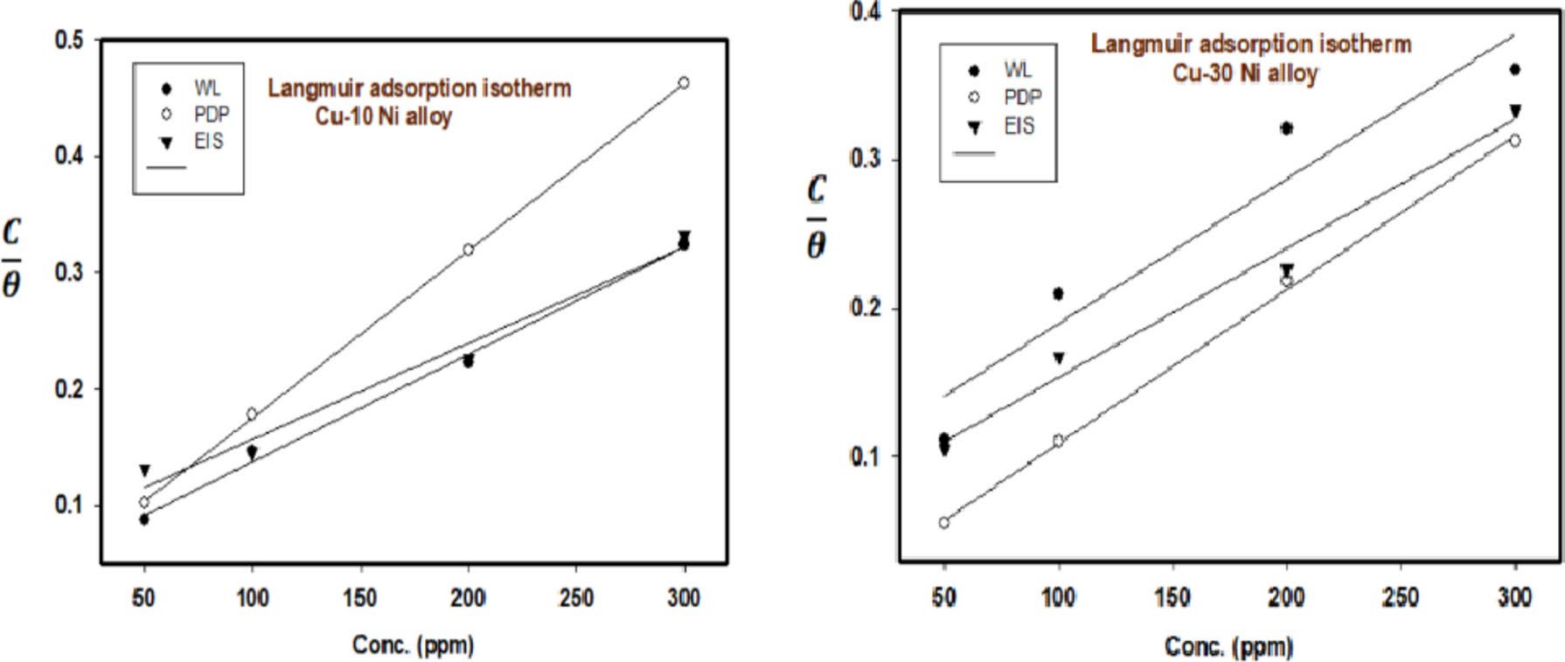
Langmuir isotherm bends for the dissolution of Cu-Ni alloys in NaCl 3.5% without and existence of altered doses of CFE at 298 K.

Figure 5 shows a plot of “C/θ on the x-axis against C on the y-axis, resembling a Langmuir adsorption isotherm. A perfect linear plot was obtained with regression constants of approximately 0.99815 and 0.9973 for Cu-10Ni and Cu-30Ni alloys, respectively. The standard Gibbs free energy of adsorption (ΔG°ads) is related to the equilibrium constant of the adsorption process and can be calculated using Eq. (7)^48^:

7 $$\:{\text{K}}_{{{\text{ads}}}} = 1/55.5\,\exp \,\left( { - \Delta {\text{G}}_{{{\text{ads}}}}^{^\circ } /{\text{RT}}} \right)$$

We calculated the standard Gibbs free energy of adsorption (ΔG°_ads_) of the inhibitor at 298 K and tabulated the results in Table 5. “When ΔG°_ads_ is around − 20 kJ/mol. or lower, the adsorption is due to electrostatic interactions between the charged inhibitor molecules and the charged electrode (physisorption). Values more negative than-40 kJ/mol. indicates charge transfer from the inhibitor to the alloy surface, forming a coordination bond (chemisorption)^49^. In this study, the observed adsorption is a combination of physisorption and chemisorption, likely because the inhibitor contains various chemical compounds ((Gum, polyphenols (phenolic acid, flavonoids, tannins) and protein)) that can be adsorbed through both mechanisms^50^. The obtained negative values of ΔG°_ads_ (less negative than − 20 kJ/mol.) suggest that the adsorption process of the inhibitors on Cu-Ni alloys in 3.5% NaCl solution is spontaneous and follows a physisorption mechanism. Table 5 shows that the Langmuir adsorption isotherm provides the best fit with high correlation coefficients (R), compared to the Temkin and Freundlich isotherms”. The values of the adsorption constant (K_ads_) obtained from the Langmuir isotherm are in good agreement with those obtained from the other two adsorption isotherms.

**Table 5:** Values of parameters from adsorbed CFE on two Cu-Ni alloys liquefaction in NaCl solution.

Isotherm	Corros. Tech.	Cu-10Ni				Cu-30Ni			
		Slope	K_ads_	R^2^	-∆G^o^_ads_	slope	K_ads_	R^2^	-∆G^o^_ads_
Langmuir	WL	9.209 × 10^− 4^	21.997	0.9953	17.61	9.754 × 10^− 4^	10.871	0.9221	15.86
	PDP	1.432 × 10^− 3^	30.713	0.9999	18.44	1.032 × 10^− 3^	15.305	0.9986	16.71
	EIS	8.240 × 10^− 4^	13.323	0.9746	16.37	8.696 × 10^− 4^	14.932	0.9843	16.65
Temkin	WL	0.488	0.267	0.9684	6.68	0.470	0.069	0.8437	3.33
	PDP	0.212	0.207	0.9881	6.05	0.065	1.083	0.6943	10.15
	EIS	0.684	0.033	0.9412	1.45	0.603	0.029	0.9506	1.15
Freundlich	WL	0.286	0.138	0.9729	19.00	0.337	0.207	0.8871	6.05
	PDP	0.163	0.234	0.9798	6.35	0.030	1.000	0.7005	9.95
	EIS	0.482	0.076	0.8967	3.57	0.390	0.527	0.9605	8.36

### Impact of temperature on inhibition process

Tables 6 and 7 summarize the corrosion rates of “Cu-Ni alloys in 3.5% NaCl solution as a function of temperature, both with and without 300 ppm carob fruit extract (CFE), as measured using potentiodynamic polarization (PDP). The addition of CFE reduces the corrosion rate at all temperatures studied. As shown in Fig. 8, the inhibition efficiency decreases slightly with increasing temperature”. To calculate the activation thermodynamic parameters of the corrosion process, we used the Arrhenius equation (Eq. (8)) ^51^.

8 $$\:{C}_{R}=A\:exp\left(\frac{{-E}_{a}}{RT}\right)\:\:\:\:\:\:\:\:\:\:\:\:\:\:\:\:\:\:\:\:\:\:\:\:\:\:\:\:\:\:\:\:\:$$

**Table 6:** Effect of temperature on electrochemical parameters of Cu-10Ni alloy in 3.5% NaCl with and without 300 ppm CFE.

Inhibitor	Temp., ^o^C	-E_corr,_ mV vs. SCE	I_corr_ µA/cm^2^	β_a_ mV/dec	- β_c_ mV/dec	CR µm/y	% IE
Blank	25	292.6	35.8300	97.6	209.0	419.0	**–**
	50	339.1	120.1888	104.2	141.2	1405	
	60	327.8	142.0984	157.9	169.1	1662	
CFE	25	281.6	12.56	92.1	155.9	146.9	64.94
	50	297.4	17.34	89.8	193.3	202.8	85.57
	60	320.9	2.161	95.8	163.1	2.522	91.48

**Table 7:** Effect of temperature on electrochemical behavior of Cu-30Ni alloy in 3.5% NaCl with and without 300 ppm CFE.

Inhibitor	Temp., ^o^C	-E_corr,_ mV vs. SCE	I_corr_ µA/cm^2^	β_a_ mV/dec	- β_c_ mV/dec	CR µm/y	% IE
Blank	25	358.3	25.3780	122.3	157.1	296.8	**–**
	50	344.2	42.2094	99.7	129.6	493.6	
	60	333.2	46.5188	62.8	88.4	544.0	
CFE	25	287.6	9.0157	31.5	51.1	11.87	65.03
	50	308.5	5.1233	56.4	88.2	59.92	87.86
	60	331.1	2.3853	74.6	105.5	74.68	94.87

**Fig. 6:**
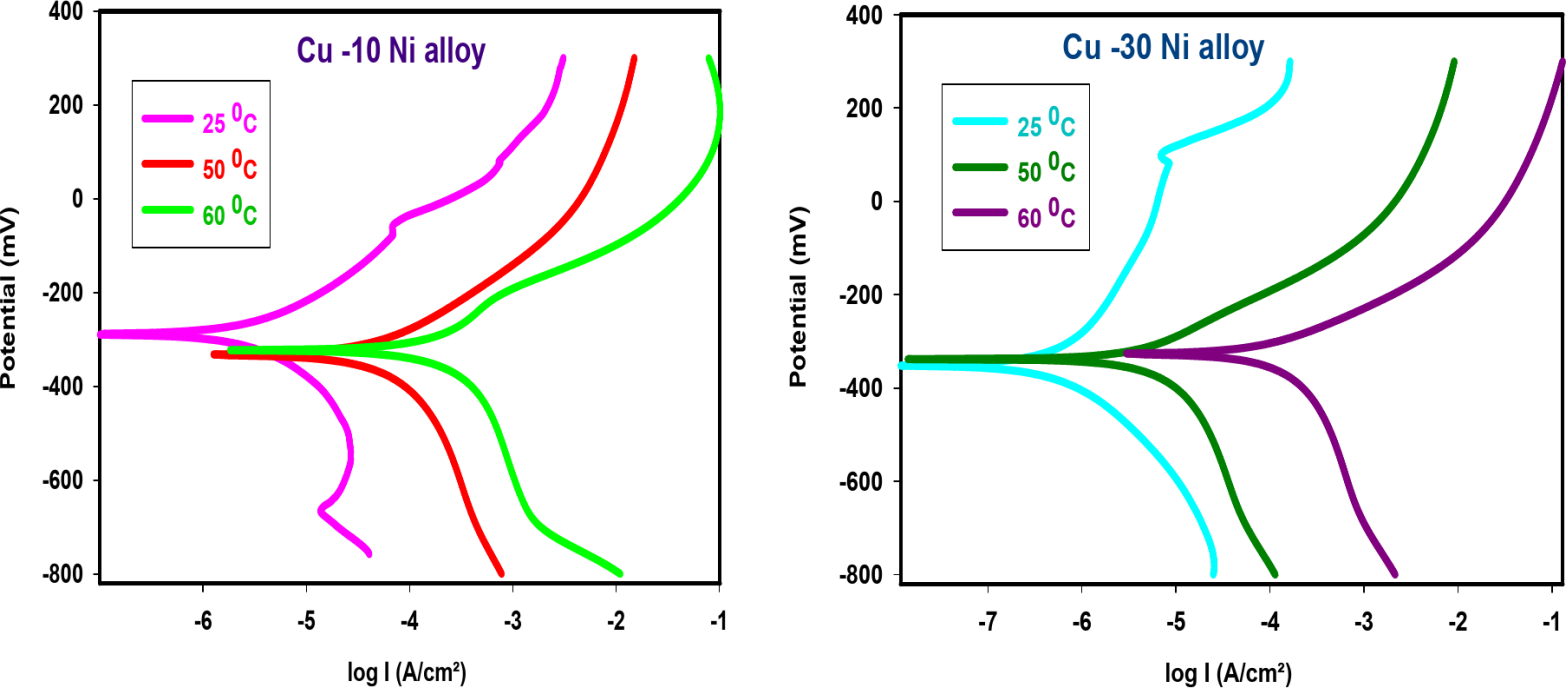
PDP bends for Cu-Ni alloys liquefaction in NaCl (3.5%), at altered temperatures.

**Fig. 7:**
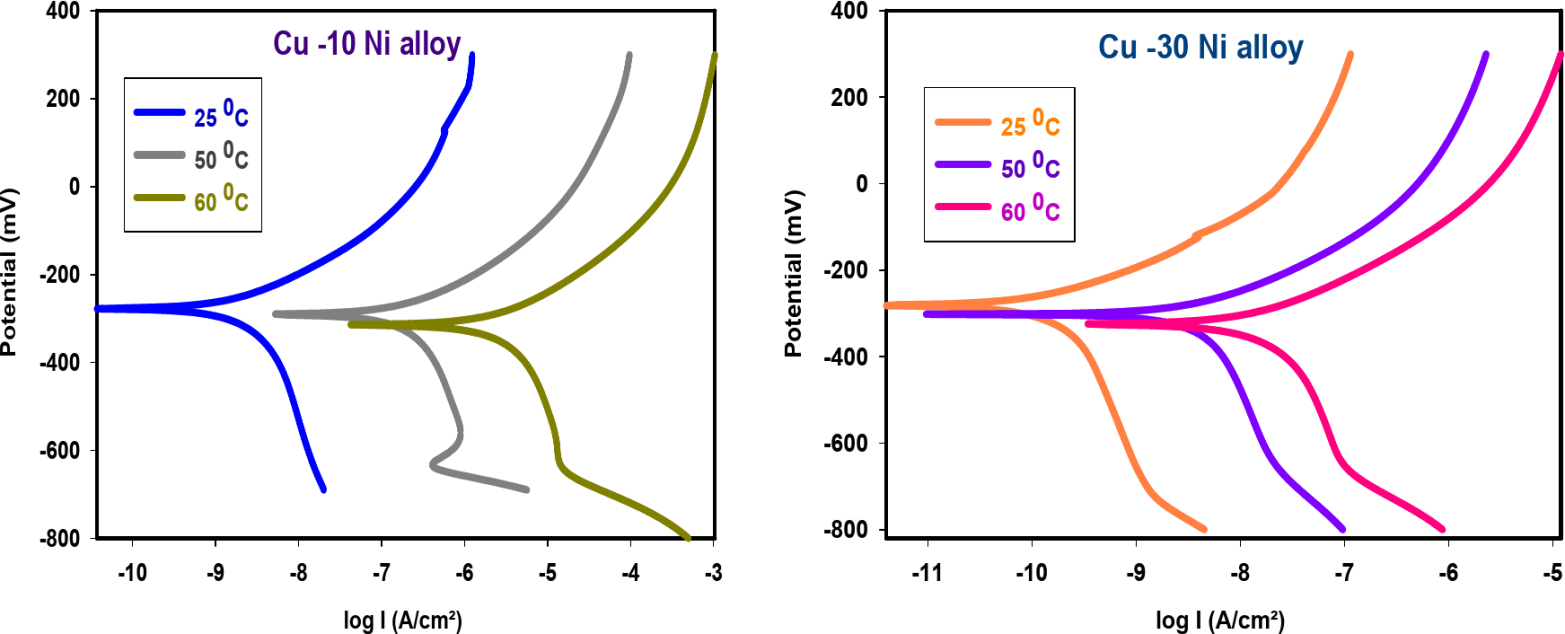
PDP bends for Cu-Ni alloys liquefaction in NaCl (3.5%) with 300 ppm CFE at different temperatures.

**Fig. 8:**
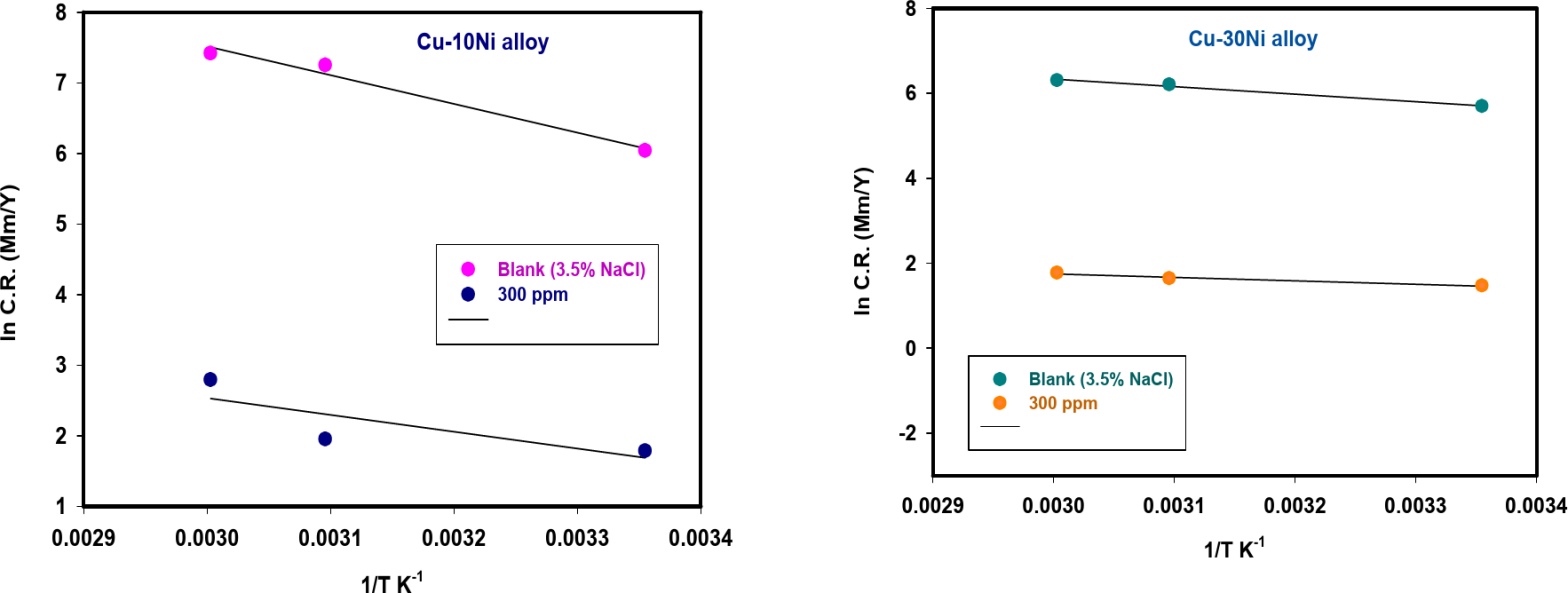
ln CR versus 1/T for Cu-Ni alloys liquefaction in 3.5% NaCl containing 300 ppm CFE at different temperatures.

**Fig. 9:**
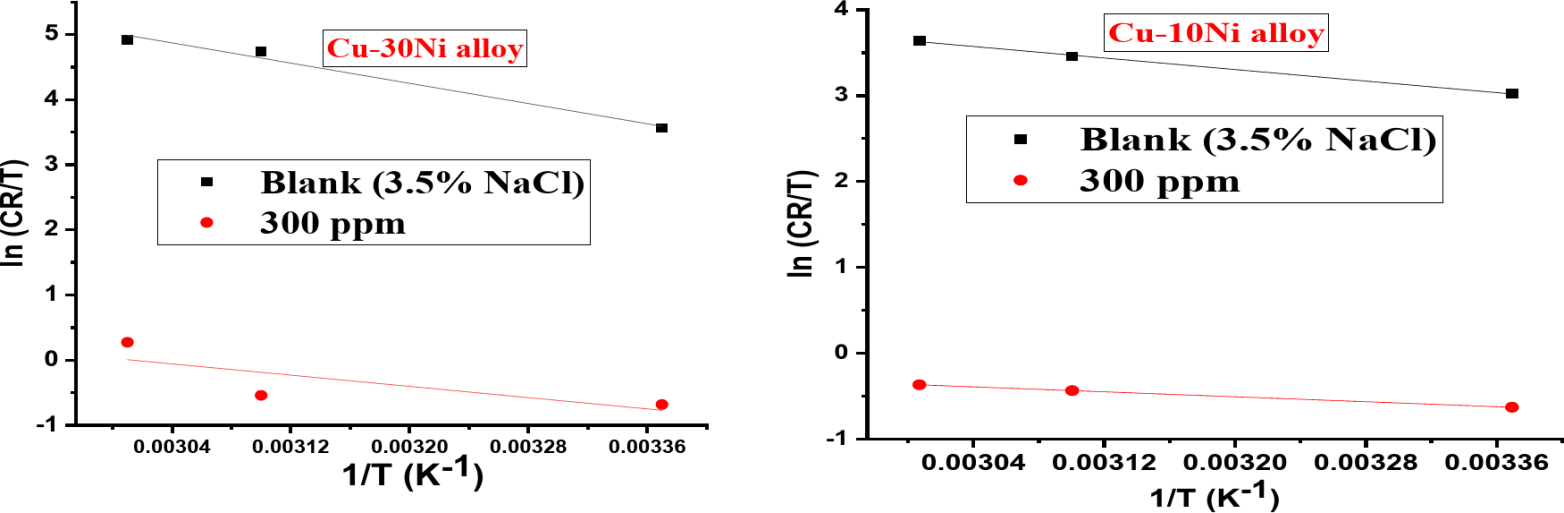
A plot of ln CR/T versus 1/T for Cu-Ni alloys in 3.5% NaCl containing 300 ppm CFE at different temperatures.

Figures 6 and 7 show the anodic and cathodic potentiodynamic polarization curves for the two Cu-Ni alloys in 3.5% NaCl solution at 25, 50, and 60°C, both in the absence and presence of 300 ppm carob fruit extract (CFE). “Tables 6 & 7 summarize the electrochemical parameters, including corrosion potential (E_corr_), corrosion current density (I_corr_), anodic and cathodic Tafel slopes (β_a_ and β_c_), and corrosion rate (CR), for Cu-10Ni and Cu-30Ni alloys, respectively. As shown in these tables, the corrosion current density (I_corr_) for both Cu-Ni alloys increases with increasing temperature, indicating that the corrosion process accelerates at higher temperatures. Conversely, the inhibition efficiency (% IE) decreases with increasing temperature. Figure 8 shows the Arrhenius plots of ln CR versus 1/T for Cu-Ni alloys in 3.5% NaCl solution at different temperatures. The slopes of these plots, -E^*^_a_/R, were used to calculate the activation energy values. Table 8 lists the calculated activation energy values and regression coefficients for Cu-Ni alloys in 3.5% NaCl solution in the presence of 300 ppm CFE. The activation energy (E^*^_a_) of an electrochemical process represents the energy barrier that electrons must overcome to transfer across the electrode/electrolyte interface. The increase in activation energy with the addition of CFE than its absence indicates a strong inhibitive action of the extract by increasing the energy barrier for the corrosion process on the Cu-Ni alloys at higher temperatures^52^. Other researchers have interpreted this variation as the formation of a physical/electrostatic adsorption film^53^.Arrhenius plots demonstrate that a higher activation energy (E^*^_a_) corresponds to a faster dependence of corrosion rates on temperature. The observed increase in E^*^_a_ suggests the formation of an adsorbed film on the alloy surface, which increases in thickness and hinders the dissolution of Cu-Ni alloys. The values of enthalpy (ΔH^*^) and entropy (ΔS^*^) were determined from the slope and intercept of Fig. 9 and are listed in Table 8”. It is evident from the Table that the activation parameters (ΔH^*^ and ΔS^*^) of alloys dissolution reaction in NaCl 3.5% in the presence and absence of CFE are higher than in the absence of extract. The positive sign of ΔH^*^ reflects the endothermic nature of the alloy dissolution process^54^. Whereas the large and negative values of entropy of activation in the absence and presence of CFE implies that the rate-determining step for the activated complex is an association step rather than a dissociation step, meaning that during adsorption process the decrease in disordering takes place on moving from reactants to the activated complex^55^. Thus ordering increases as reactants are converted to activate complex. “The findings are consistent with the well-understood thermodynamic relationship between activation energy (E^*^_a_*)* and enthalpy (ΔH^*^) (Table 8) as shown in Eq. 9.

9 $${\text{E}}_{{\text{a}}}^{{\text{*}}} - \Delta {{\text{H}}^*}={\text{RT}}$$

“The reduction in entropy indicated by the negative ΔS^*^ with CFE points to a more ordered state. This could be due to the binding of extract molecules to the alloy surface, displacing water molecules from the electrode surface^56^.

**Table 8:** Values of E_a_^*^, ΔH^*^ and ΔS^*^ for Cu-Ni alloys in corrosive medium in existence and lack of 300 ppm CFE.

Alloy	E_a_*	ΔH*	-ΔS*	*R* ^2^
Cu-10Ni (Blank)	19.827	17.345	92.3	0.9790
Cu-10Ni + 300 ppm	35.758	33.198	168.2	0.9895
Cu-30Ni (Blank)	6.772	4.248	25.1	0.9653
Cu-30Ni + 300 ppm	15.604	13.124	85.7	0.9749

### SEM/EDX investigation

**Fig. 10:**
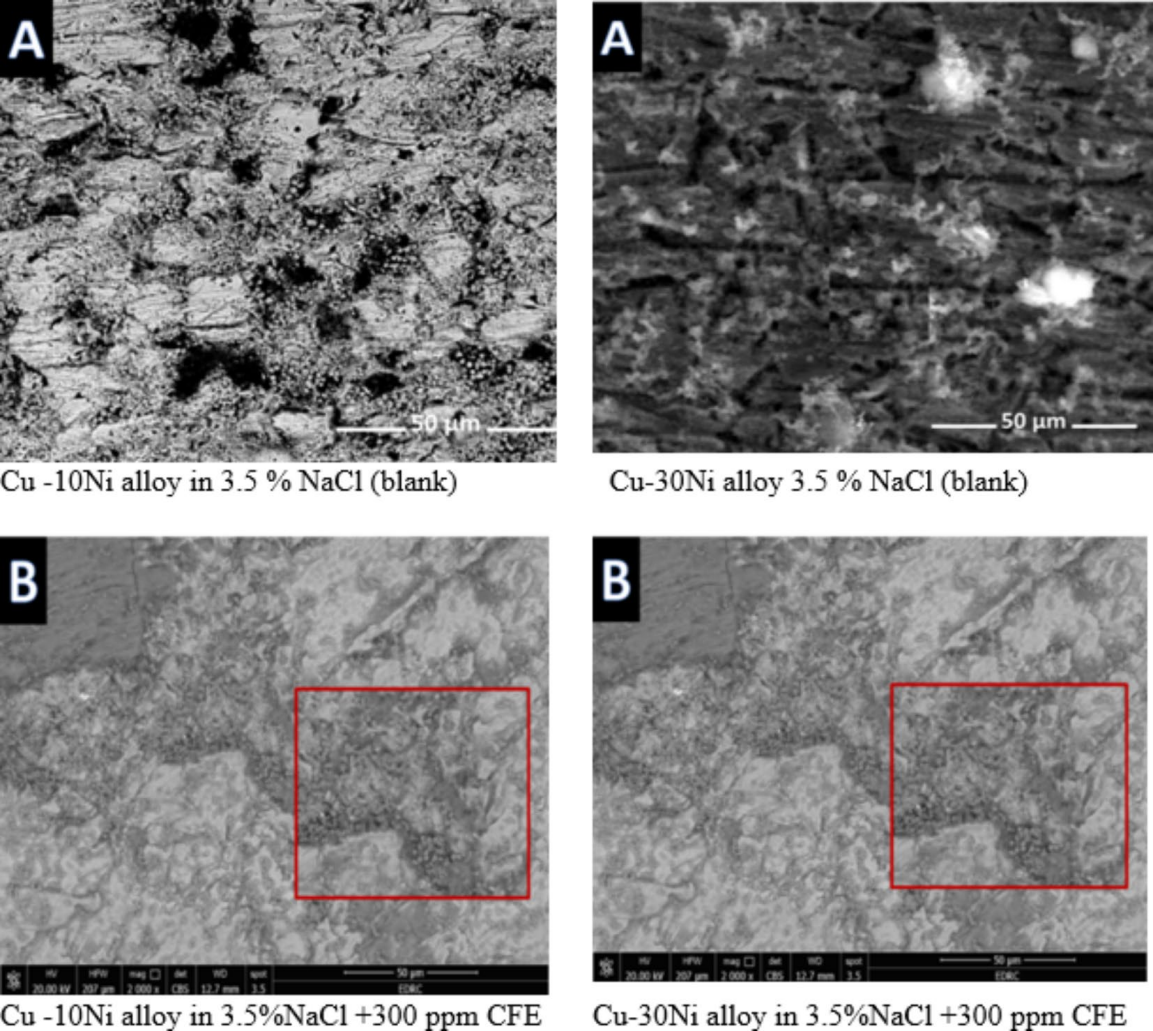
Surface morphology for Cu-Ni alloys exposed to 3.5 % NaCl solution with 300 ppm CFE.

**Fig. 11:**
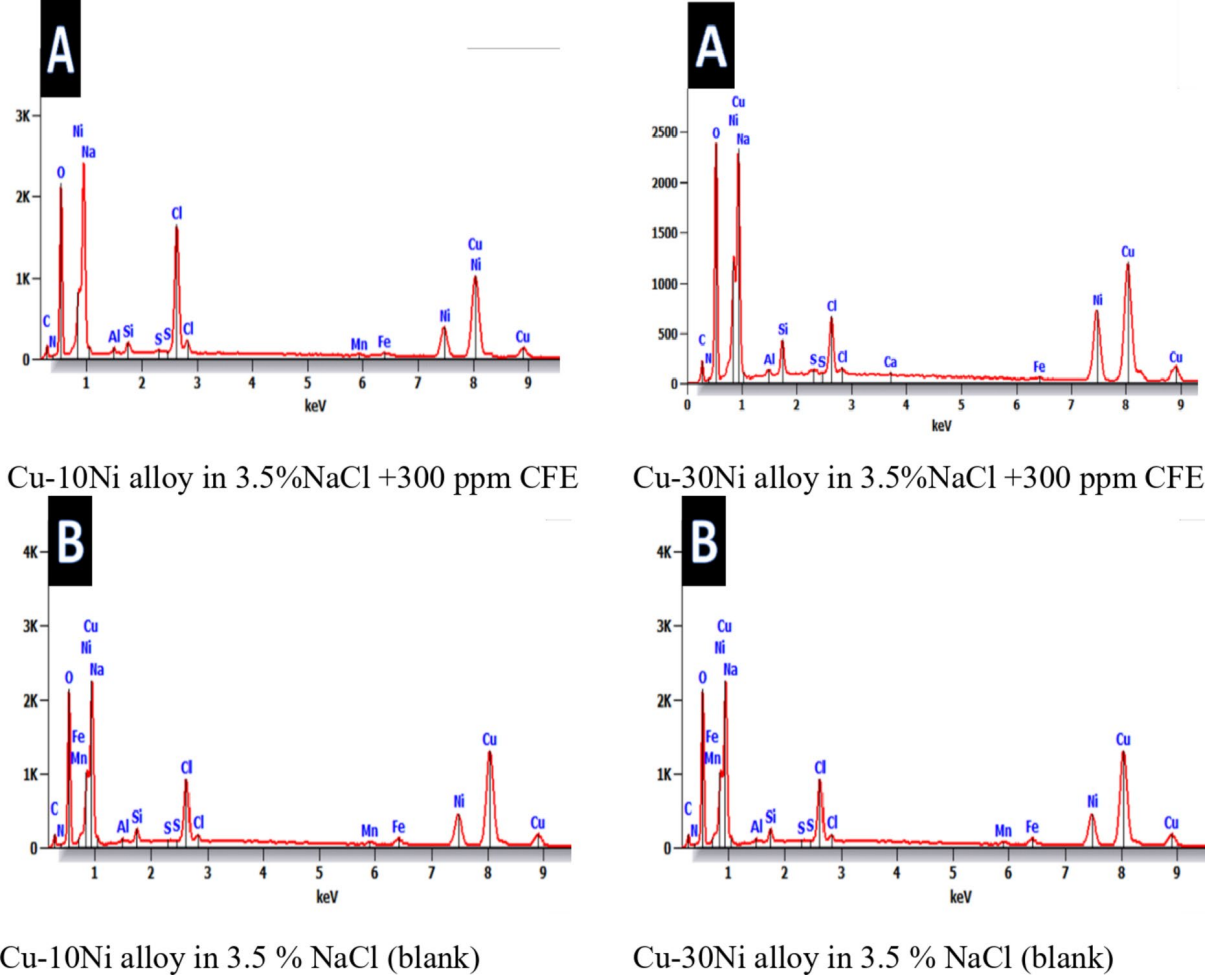
EDX spectra for the Cu-Ni alloys exposed to 3.5 % NaCl solution with 300 ppm CFE.

Figure 10 obviously illustrates the SEM images of Cu-Ni alloys exposed to 3.5% NaCl solution with and without inhibitor. The SEM image of the corroded Cu-Ni alloy after submersing in 3.5% NaCl solution and which appeared as a rough surface with many cracks and pores. This result is due to the Cl- ions in the medium aggressively attacking the electrode surface, causing surface corrosion. On the other hand, in the presence of CFE, less destruction was observed in the micrographs of the electrodes. These results confirm that the surface has greatly enhanced, with a valuable and observable reduction in the corrosion rate. This improvement is owing to the creation of a good protective layer on the Cu-Ni alloys surface from the inhibitor Carob, which prevents the corrosion. Figure 11 shows the corresponding energy-dispersive X-ray (EDX) spectra of the surfaces of two Cu-Ni alloys. This figure reveals the elements present on the alloy surfaces. The analysis indicates that the predominant species on the electrode surfaces is the adsorbed protective layer. EDX analysis (Table 9) of the surface with CFE extract shows the presence of oxygen and a reduction in the chloride signal. This suggests that the extract adsorbs onto the Cu-Ni alloy surface through oxygen-containing active centers. Additionally, the less porous nature of the inhibitor-covered surface may hinder the penetration of chloride ions into the Cu-Ni alloys.

**Table 9:** Surface composition (mass %) of Cu-Ni alloys before and after 3 h of exposure to corrosive medium without and with 300 ppm of Carob fruit extract.

Samples	Mass %							
	O	Na	Cl	Mn	Fe	Ni	Cu	Si
Free Cu-10 Ni alloy	17.2	1.1	10.2	0.6	1.1	14	51.8	–
In presence of 300 ppm extract	10.9	0.8	1.9	0.6	1.4	7.7	68.9	–
Free Cu-30 Ni alloy	16.4	0.7	3.3	0.1	0.4	23.9	51.1	1.7
In presence of 300 ppm extract	14.6	0.5	2.5	0.1	0.2	24.6	56.4	0.7

### AFM analysis

**Fig. 12:**
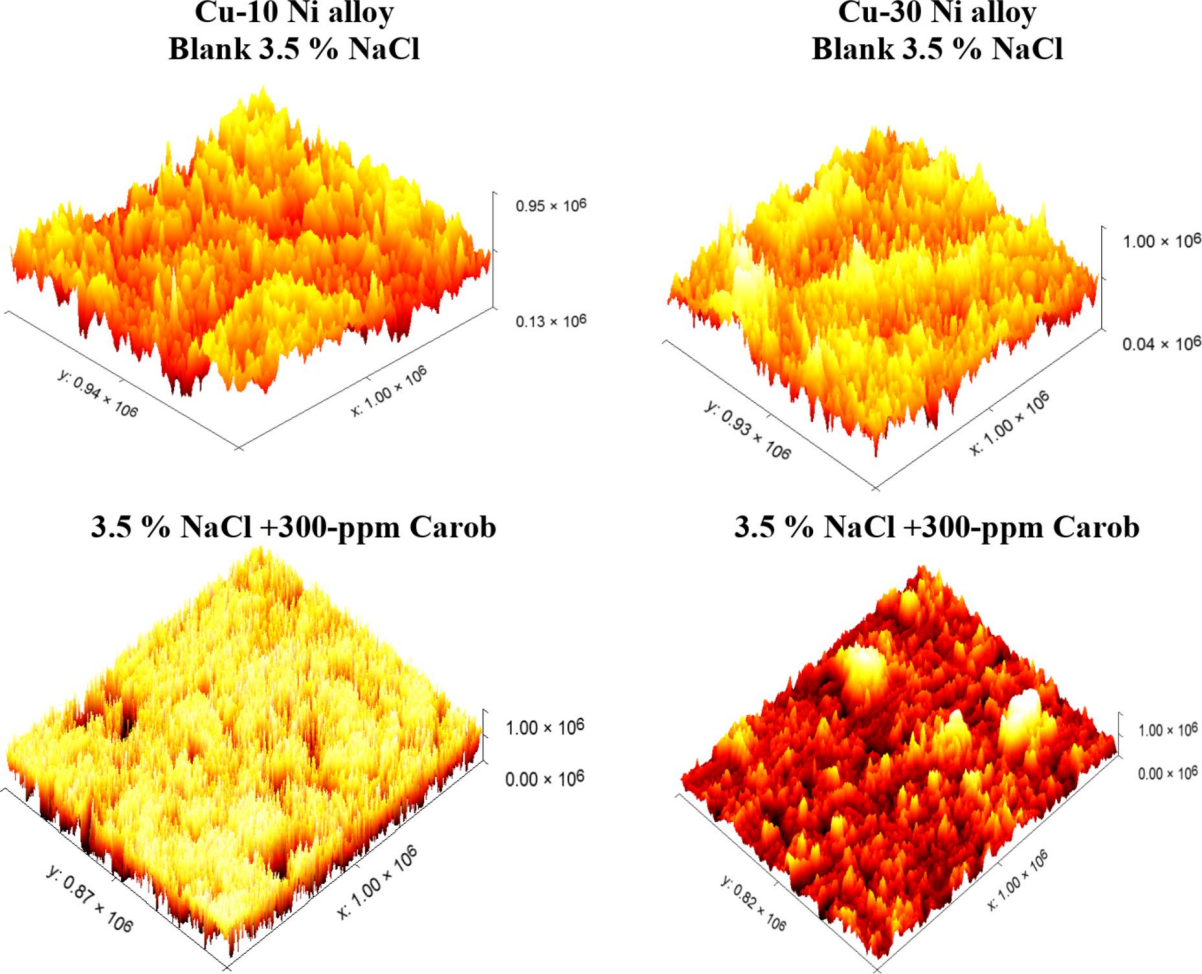
AFM micrograph for Cu-Ni alloys in 3.5 % NaCl without (a) and with 300 ppm of extract.

To investigate the existence of an inhibitor film on the Cu-Ni alloy surfaces, we conducted atomic force microscopy (AFM) analysis. “Figure 12 shows the AFM images and force curves obtained after 24 hours of exposure to 3.5% NaCl solution with and without the corrosion inhibitor. The mean roughness value of the Cu-10Ni alloy surface exposed to 3.5% NaCl solution without the inhibitor was significantly higher at 300 nm compared to the Cu-30Ni alloy, which had a mean roughness of 400 nm. The corrosive effects of the acid over the 24-hour rust test period left the Cu-Ni alloy surfaces with a porous structure and deep fractures, leading to this increased roughness. However, when the inhibitor was applied at the optimal dose of 300 ppm, the average roughness for Cu-10Ni and Cu-30Ni alloys was reduced to 81 nm and 105 nm, correspondingly”. This suggests that the inhibitor effectively maintains the hardness of the Cu-Ni alloys, as evidenced by the decrease in roughness value ^57,58^.

## Conclusions

Carob Fruit, a naturally occurring substance, has demonstrated its effectiveness as a green corrosion inhibitor for Cu-Ni alloys in marine environments. “Cu-Ni alloys corrosion rate and inhibition competency are evaluated by the WL, PDP and EIS tests in NaCl (3.5%). According to WL tests, the rates of corrosion decreased as the dose of CFE increased. For Cu-10Ni and Cu-30Ni alloy, the corresponding CR values are 1.6404 and 0.9482 g cm^− 2^ day^− 1^, respectively. With 300-ppm CFE, the % IE from EIS was ~ 81.5% for Cu-10 Ni alloy and ~ 83.4% for Cu-30 Ni alloy, correspondingly. The CR and I_corr_ decrease, while the resistance and IE increase with increasing CFE dose. Polarization data revealed that Carob Fruit extract behave as mixed type inhibitor for both alloys. The adsorption of the CFE follows Langmuir, Temkin, and Freundlich isotherm models. The calculated values of ΔG°_ads_ and E^*^_a_ suggest that the adsorption mechanism is physisorption, but by increasing temperature the % IE increase indicate that the adsorption is chemical, so the adsorption of CFE on Cu-Ni alloys is of mixed type. The results obtained from SEM and EDX spectra confirm the presence of a protective film on the alloy surface from CFE molecules. In the future one can use this extract as green corrosion inhibitor for metals such as steel, copper, aluminum, zinc and also other alloys in different acidic media and in NaCl polluted solution.

## Electronic supplementary material

Below is the link to the electronic supplementary material.

Supplementary Material 1

## Data Availability

The authors confirm that the data supporting the findings of this study are available within the article [and/or] its supplementary materials.
