# Supplementary material for: Carob fruit extract as naturally products corrosion inhibitor for copper-nickel alloys in brine solutions
Source: Sci Rep. 2024 Nov 26;14:29290. doi: 10.1038/s41598-024-80589-7 (PMC11599866; doi:10.1038/s41598-024-80589-7)
Supplement: Supplementary file 1 — Supplementary Material 1 [file 41598_2024_80589_MOESM1_ESM.docx]

| 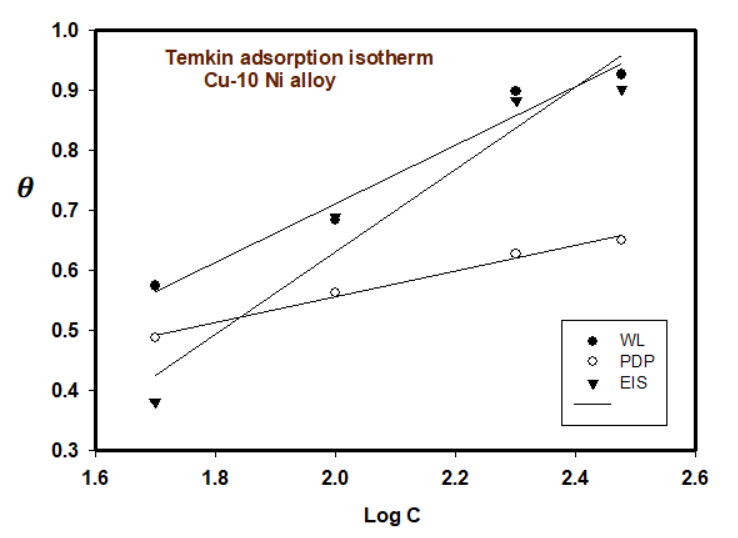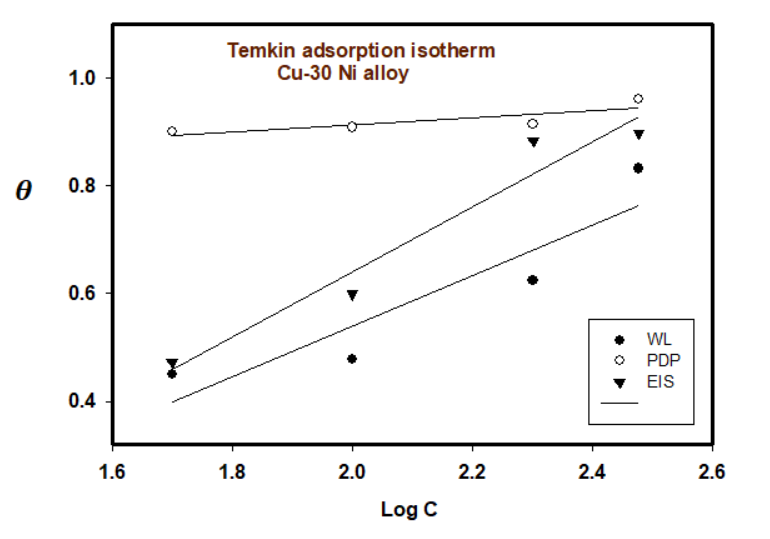 |
| --- |
|  |

**Carob Plant Extract as Naturally Products Corrosion Inhibitor for Copper-Nickel Alloys in Brine Solutions**

**Abd El Aziz S. Fouda^1*^, Mona Nageeb^2^, Mohamed F. Atia^3^, Ghalia A. Gaber^2^**^*^**, Amal S. I. Ahmed^2^, Ahmed A El-Hossiany^1,4^**

**^1^**Chemistry Department, Faculty of Science, Mansoura University, Mansoura -35516, Egypt

Email: asfouda@mans.edu.eg

**^2^**Department of Chemistry, Faculty of Science (Girls), Al-Azhar University, P.O. Box: 11754, Yousef Abbas Str., Nasr City, Cairo, Egypt

^3^Institute of Aviation Engineering and Technology, Cairo, Egypt.

^4^ Delta for Fertilizers and Chemical Industries, Talkha, Egypt.

**Fig. S1.** Temkin isotherm plot for the corrosion of Cu-Ni alloys in 3.5 % NaCl solution in the absence and presence of different doses of Carob at 25^o^C

| 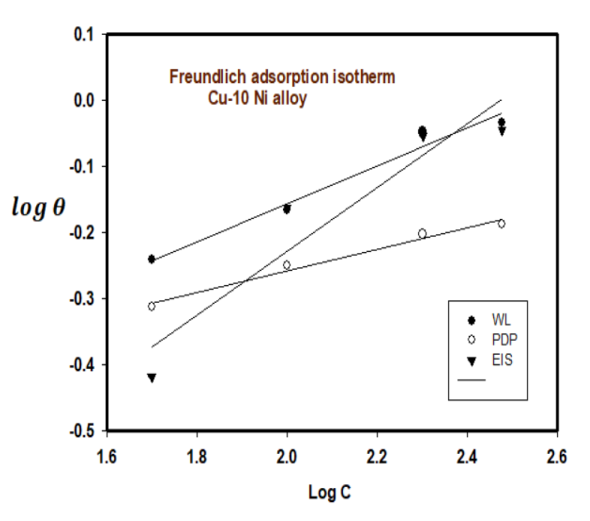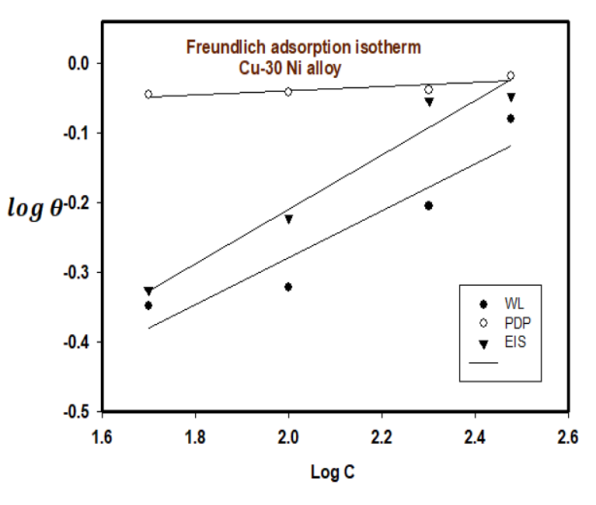 |
| --- |
|  |

**Fig. S2.** Freundlich isotherm plot for the corrosion of Cu-Ni alloys in 3.5 % NaCl solution in the absence and presence of different doses of Carob at 25^o^C

**Temkin adsorption isotherm**

The surface coverage (θ) is connected to the inhibitor dose (C) and the adsorption equilibrium constant K_ads_ as shown in Eq. 1 [37].

Exp ^(−2a θ)^ = K_ads_ × C (1)

where a is the attractive parameter and K is the adsorption equilibrium constant. From Fig. S1 linear plots are obtained, which affirms that the adsorption obeys the Temkin adsorption isotherm. Adsorption parameters obtained from this Figure are shown in Table S1.

**Freundlich adsorption isotherm**

According to the Freundlich isotherm, θ is related to the inhibitor dose C by Eq. 2 [38].

log θ = log K_ads_ + nlog C (2)

where n is the empirical constant, and the other constants have the same meaning. Fig. S2 shows straight lines relation of log θ against log C with slope n and intercept log K_ads_. The deduced adsorption parameters K_ads_, n, and ΔG^0^_ads_ are shown in Table S1. The obtained values of the correlation factor are far from unity. The adsorption process was studied using Langmuir, Freundlich, and Temkin isotherms. The adsorption studies clearly indicated that the experimental data satisfied the Langmuir Freundlich, and Temkin adsorption isotherms with good linearity. The chosen criteria of the best-fit isotherm are based on the higher correlation coefficient. The higher value of K_ads_ indicates that the inhibitor is strongly adsorbed on the Cu-Ni surface.

**Table S1.** Values of parameters of Langmuir adsorption isotherm for two Cu-Ni alloys with the addition of Carob in 3.5 % NaCl solution

| **Isotherm** | **Corros Tech.** | **Cu-10Ni** | | | | **Cu-30Ni** | | | |
| --- | --- | --- | --- | --- | --- | --- | --- | --- | --- |
|  |  | **slope** | **K_ads_** | **R^2^** | **-∆G^o^_ads_** | **slope** | **K_ads_** | **R^2^** | **-∆G^o^_ads_** |
| Langmuir | **WL** | 9.209x10^-4^ | 21.997 | 0.9953 | 17.608 | 9.754x10^-4^ | 10.871 | 0.9221 | 15.86 |
|  | **PDP** | 1.432x10^-3^ | 30.713 | 0.9999 | 18.436 | 1.032x10^-3^ | 15.305 | 0.9986 | 16.71 |
|  | **EIS** | 8.240x10^-4^ | 13.323 | 0.9746 | 16.366 | 8.696x10^-4^ | 14.932 | 0.9843 | 16.65 |
| **Temkin** | **WL** | 0.488 | 0.267 | 0.9684 | 6.680 | 0.470 | 0.069 | 0.8437 | 3.33 |
|  | **PDP** | 0.212 | 0.207 | 0.9881 | 6.046 | 0.065 | 1.083 | 0.6943 | 10.15 |
|  | **EIS** | 0.684 | 0.033 | 0.9412 | 1.445 | 0.603 | 0.029 | 0.9506 | 1.15 |
| **Freundlich** | **WL** | 0.286 | 0.138 | 0.9729 | 19.003 | 0.337 | 0.207 | 0.8871 | 6.05 |
